# Introduction: development of the sterile insect technique for African malaria vectors

**DOI:** 10.1186/1475-2875-8-S2-I1

**Published:** 2009-11-16

**Authors:** Waldemar Klassen

**Affiliations:** 1 Tropical Research and Education Center, University of Florida, Homestead, Florida 33031, USA

## 

This supplement to *Malaria Journal *meets a great need for a convenient assemblage of existing information on the suppression and/or eradication of *Anopheles *populations using the release of sterilized mosquitoes. Publication of such a collection of articles is overdue for three compelling reasons. Firstly, because malaria control in sub-Saharan Africa, where 90 percent of the 300 to 500 million malaria cases and one to three million deaths occur from malaria each year, still depends on only two technologies for vector intervention: indoor residual spraying and insecticide-treated bed nets. Secondly, considerable research and development on the suppression of mosquitoes with the sterile insect technique (SIT) was conducted from the mid-1950s to the mid-1970s. However, nearly all of the scientists who pioneered this approach have retired and several of the greatest have died. While the benefit of the input, judgement and guidance can be provided from current experts in this field, a record of the key contributions of people like Chris Curtis, Ed Knipling and Don Weidhaas has thus far not been assembled. Thirdly, there are now new technologies available to support area-wide integrated pest management (AW-IPM) programmes and much experience has been gained with the implementation of these programmes against major insect pests that could be applied to mosquito control [1,2].

In Europe, north of the Alps and Pyrenees, and in the United States, malaria began to decline in the mid 1800s as a result of drainage of swamps, use of screens to exclude insects on windows and doors, increased availability of quinine, increased literacy and improved education and general increase in economic well-being [3]. Endemic malaria in the USA largely disappeared without the eradication of the anopheline vectors in the late 1940s, when malaria cases were aggressively treated and large areas sprayed with DDT. Eradication of endemic malaria in the USA probably would have been accomplished even without DDT.

Malaria is transmitted only by mosquitoes in the genus *Anopheles *and in sub-Saharan Africa, most transmission from infected to healthy people is accomplished by three anopheline species, *Anopheles arabiensis*, *Anopheles funestus *and *Anopheles gambiae*, with *An. gambiae *being the most important. *Anopheles gambiae *was eradicated in Brazil in 1940 [4] and from Egypt in 1945 [5]. Also, because most mosquitoes readily enter human dwellings and rest on walls and ceilings, it was found that they could be killed through applications of residual insecticides to these surfaces. Thus, in the 1930s de Meillon in South Africa had shown that malaria could be controlled by frequent spraying of the walls and ceilings of dwellings with pyrethrins [6], and this was substantiated by work in India [7]. The effectiveness of such residual treatment was found to be vastly prolonged by the use of DDT [8]. Residual treatment was found to result in the complete interruption of transmission in Cyprus, Greece, Italy, Sardinia, Taiwan, Venezuela and the USA. These historic achievements induced confidence that worldwide eradication of malaria through vector control was feasible. Thus, in 1955, the World Health Assembly launched the Global Malaria Eradication Programme with applications of DDT within dwellings being the primary stratagem [9].

Eradication of malaria was accomplished in due course in the temperate areas of Europe and Asia, in some subtropical areas, the Mediterranean Basin, and the southern USA, and on a number of the tropical islands of the Caribbean [8], and malaria was greatly reduced in Brazil and India. Spectacular progress was made in Sri Lanka, where the number of cases was reduced from a high of two or more million cases each year in 1958 to just 17 in 1963. In 1964 spraying was halted and the consolidation phase of the Programme was implemented. Meanwhile, newly discovered gem fields attracted large numbers of miners into the previously malaria endemic area who dug large numbers of pits in search of gem-stones. The soon-abandoned pits filled with water and became the source of dense populations of *Anopheles culicifacies*, the main malaria vector [10]. In 1967 malaria resurged strongly and spraying was resumed, but the dense vector population was found to have become resistant to DDT. Malathion was substituted, but it was objectionable to some families, required more frequent applications and was more expensive. Thus, the battle was lost and the incidence of malaria grew to more than 500,000 cases each year [3].

As described above, some economic development has diminished transmission, but certain ecological changes, which attend economic development, strongly favour the population growth of *Anopheles *species. In particular, water development projects (dam construction, expanded irrigation, production of wetland rice) tend to create ideal mosquito breeding conditions, while mining, industrialization, urbanization and deforestation also tend to shift ecological conditions in favour of growth of vector populations and malaria transmission [10].

By 1966, progress in the eradication of malaria had slowed noticeably, and in 1967 the World Health Assembly retreated from the goal of global eradication. Thus, the people of sub-Saharan Africa failed to benefit from the Global Malaria Eradication Programme (1957-1967). Nevertheless, the World Health Organization (WHO) conducted numerous pilot projects in sub-Saharan Africa to assess the degree of effectiveness of the residual treatment approach alone and in conjunction with mass administration of anti-malarial drugs. WHO coordinated a particularly thorough large-scale pilot project in the Garki District of northern Nigeria from 1969 to 1976. This study showed that in certain villages, the intensity of transmission and the vectorial capacity sometimes approached up to 200 times the critical value for interruption of transmission and the number of infectious bites per person per annum (the so-called Entomological Inoculation Rate) reached 132 in one village during the 6-month rainy season. Even though residual insecticide treatment reduced vectorial capacity by 90%, the incidence of malaria was reduced only by 25%. Immigration of vectors and humans from untreated sites was not considered to have been a significant factor in causing such unsatisfactory suppression of malaria. The primary reason for the lack of high efficacy of the residual treatment approach was found to be the tendency of the vectors to rest outdoors after blood feeding (exophily). Even the addition of mass drug administration at frequent intervals with strong community participation failed to halt transmission, although it did reduce the incidence of malaria to very low levels. Thus in such areas where ecological conditions support extremely high vector populations, the combination of residual treatment and drug administration cannot halt transmission.

The inadequacy of area-wide residual treatment can be overcome to a significant degree by the area-wide use of insecticide-impregnated bed nets. While impregnated bed nets cannot protect against outdoor feeding of a significant fraction of the vector population [2], they do prevent a significant fraction of the vector population from attaining the age of about 12 days - the age at which the females can become infective [11].

Since malaria transmission can be interrupted only if the density of the vector population is reduced below a critical level, determined efforts were made from the mid-1950s to the mid-1970s to develop the SIT for mosquito suppression. Edward F Knipling, the inventor of the technique, advanced the hypothesis that if sterile males are released initially at a rate (number per km^2^) high enough to cause a decline in the wild population, then the sustained release of this constant number of sterile males into the wild population each generation will achieve a progressively greater degree of suppression of the wild population in each successive generation [12]. In contrast, an insecticide at a constant dosage rate tends to kill the same fraction of an insect population at all population densities. Further, Knipling posited that the SIT could be integrated with the use of cultural, biological and chemical measures to achieve robust and powerful systems of population control. The initial reduction of the vector population by, say, 90-98 percent, would greatly reduce the number of sterile males needed to achieve a continuing downward trend in the population. *The SIT, other genetic methods and sex-pheromone traps are the only methods known to science, which become progressively more powerful with decreasing population density of the target pest*.

A number of successful small-scale field trials were conducted with one or more species each of *Aedes, Culex *and *Anophele*s [13]. The largest-scale trials were conducted in El Salvador and India. Unfortunately, both of these trials were interrupted in the mid-1970s, before they could be completed. In El Salvador, the work was terminated by the eruption of civil war and in India by hysteria induced by false accusations that the project was intended to collect data on biological warfare.

In India, the work led by the late Chris Curtis showed that two important culicine vector species could be mass-reared, and the sexes separated (according to pupal size) to ensure that 99.8% of the released insects were males. Males were either chemosterilized in the pupal stage, or their sterility was produced by male-linked chromosome translocations combined either with cytoplasmic incompatibility or sex-ratio distortion due to meiotic drive. Field tests showed that the mating competitiveness of the males of both species was acceptable. However, the mass release of *Culex quinquefasciatus *males in villages achieved only limited levels of sterility in eggs laid by wild females because of the influx of already-mated females from outside the target release area. A planned mass release of sterile male *Aedes aegypti*, aimed at the eradication of this urban mosquito from a whole town, was prevented by the political problem mentioned above.

In El Salvador, the target was the malaria vector *Anopheles albimanus*, and the work was conducted by a team from the US Department of Agriculture Laboratory, Gainesville, Florida The wild population was multi-resistant to insecticides (partly due to the agricultural use of insecticides) and, therefore, difficult to control by conventional means. In the initial study, releases during five months around Lake Apastapeque were successful in inducing 100% sterility in eggs laid by wild females. Later, sex separation was greatly improved by a genetic sexing strain in which a chromosome translocation was induced to link a propoxur-resistance gene to the Y chromosome, and this was combined within a crossover-suppressing chromosome inversion. Propoxur treatment at the egg stage selectively eliminated all but 0.2% of females, thereby allowing a doubling of the male production for release. By eliminating the handling losses in the adult stage, the net release was increased from 200,000 males per day to over one million, and these males, when released as sterile pupae, were almost fully competitive in the field. Compared with the seasonal upward trend of the untreated population, the releases reduced the target field population by more than 97%.

The immigration of females already inseminated by fertile males outside the release area is a major obstacle to progress for AW-IPM programmes using sterile insects. In the case of the New World screwworm, *Cochliomyia hominivorax*, eradication in North America, this problem was overcome by large-scale rolling programmes of release of sterile insects. Ten years ago, Curtis and Andreasen [11] sadly asserted that "*finding the capital for this seems unlikely for a programme directed against An. gambiae, which extends over huge areas of rural Africa and threatens the lives of the children of the poor, but not cash crops which accountants see as a worthwhile investment*."

However, better times are dawning for Africa. The African Union, formed in 2002 is gradually resolving conflicts and improving social and economic conditions including commerce and international trade. African leaders in 2000 had strongly committed their governments to malaria eradication in the Abuja Declaration and in their support of the Roll Back Malaria Initiative of the World Health Organization. Africa has become a priority for bilateral and multilateral development and public health assistance. At the 1998 G8 Summit, the leaders of the world's wealthiest countries pledged to increase donor funding to combat malaria, and some of this is being provided by the European Alliance Against Malaria, the Global Fund to Fight AIDS, Tuberculosis and Malaria, the U.S. President's Malaria Initiative [14].

There is renewed interest in the scientific community to improve or even replace the SIT through the techniques of molecular biology to make *Anopheles *incapable of transmitting the *Plasmodium *protozoan parasite. Thus, IAEA seems to have been especially prescient in initiating relevant planning in 2001 and in launching a project in 2004 to assess the feasibility of the use of the SIT against selected populations of *An. arabiensis *in sub-Saharan Africa. All Africans and all who wish Africa well have a tremendous stake in the outcome. Likewise, this supplement, "Development of the Sterile Insect Technique for African malaria vectors", will become available early in this new era when the potential for beneficial impact is the greatest. The authors of the various chapters deserve our respect and high praise for assembling a supplement, which will have to be on the desk of every investigator interested in combating the vectors of malaria in Africa.

I feel deeply honoured by having been given the privilege of providing this Foreword of a series of articles that seems certain to usher in a new successful era in the epic struggle against malaria, the most deadly and persistent malady of mankind.

## Competing interests

The author declares that they have no competing interests.
